# Malaria Parasite Density Estimated with White Blood Cells Count Reference Value Agrees with Density Estimated with Absolute in Children Less Than 5 Years in Central Ghana

**DOI:** 10.1155/2015/923674

**Published:** 2015-04-07

**Authors:** Dennis Adu-Gyasi, Kwaku Poku Asante, Sam Newton, Sabastina Amoako, David Dosoo, Love Ankrah, George Adjei, Seeba Amenga-Etego, Seth Owusu-Agyei

**Affiliations:** Kintampo Health Research Centre (KHRC), Box 200, Kintampo North, Brong Ahafo, Ghana

## Abstract

*Introduction*. The estimation of malaria parasite density using a microscope heavily relies on White Blood Cells (WBCs) counts. An assumed WBCs count of 8000/*µ*L has been accepted as reasonably accurate in estimating malaria parasite densities due to the challenge to accurately determine WBCs count.* Method*. The study used 4944 pieces of laboratory data of consented participants of age group less than 5 years. The study compared parasite densities of absolute WBCs, assumed WBCs, and the WBCs reference values in Central Ghana. Ethical approvals were given by three ethics committees.* Results*. The mean (±SD) WBCs and geometric mean parasite density (GMPD) were 10500/*µ*L (±4.1) and 10644/*µ*L (95% CI 9986/*µ*L to 11346/*µ*L), respectively. The difference in the GMPD compared using absolute WBCs and densities of assumed WBCs was significantly lower. The difference in GMPD obtained with an assumed WBCs count and that of the WBCs reference values for the study area, 10400/*µ*L and 9200/*µ*L for children in different age groups, were not significant.* Discussion*. Significant errors could result when assumed WBCs count is used to estimate malaria parasite density in children. GMPD generated with WBCs reference values statistically agreed with density from the absolute WBCs. When obtaining absolute WBC is not possible, the reference value can be used to estimate parasite density.

## 1. Background

Malaria is caused by one or a combination of four species of* Plasmodia* and leads to over one million deaths, of which over 75% occur in African children under 5 years infected mainly with* Plasmodium falciparum (Pf)*.* Pf *and* Plasmodium vivax (Pv)* are identified as the* Plasmodium* species responsible for causing the most severe form of malaria [1].

In epidemiological studies, intervention studies, and clinical trials, malaria microscopy is routinely relied upon as a primary endpoint measurement of the level of malaria infection [2]. This is expressed as parasite density and is classically defined as the number of asexual forms of parasite relative to a blood volume (e.g., microliter) [3]. In the four basic counting techniques using microscopy [3], White Blood Cells (WBCs) are relatively used in estimating* Plasmodium* parasitaemia by counting the number of parasites against a predetermined number of WBCs on Giemsa stained blood smears.

Complete blood counts, particularly WBCs count, can be performed with new generation automated haematology analysers [4] and/or manually using stained microscope smears and the Neubauer chamber and counters [5, 6]. Some health facilities frequently use manual convention methods to determine the complete blood counts of a patient for management as a result of a high cost of purchasing and maintaining fully automated or semiautomated haematology analyzers. Another cost burden using an automated system includes the ability to ensure prompt validation, maintenance, and implementation of rigorous quality systems. Automated machines may therefore not be the preferred choice to quantify WBCs in resource poor areas.

Due to the frequent lack of facilities in some malaria endemic countries to quantify WBCs, an assumed WBCs count of 8000/*μ*L of blood has been accepted by World Health Organization as reasonably accurate [7] to estimate malaria parasite densities. Assumed WBCs count of blood may generate systematic errors which could produce incorrect conclusions in patient management or during clinical research that uses malaria parasite counts as an end point [3, 8].

Medical laboratories need to set relevant reference ranges for WBCs and other common laboratory parameters for clinical management of patients or during malaria clinical research [9]. Kintampo Health Research Centre has established reference values for common haematological and biochemical laboratory parameters for its study area covering an area of about 7200 km^2^ [10].

The team compared the geometric mean parasite densities (GMPD) calculated using absolute WBCs, assumed WBCs, and WBCs reference values of the participants enrolled in the malaria studies in middle Ghana to assess the impact of using these as malaria parasite density estimators.

## 2. Methods

### 2.1. Ethics Statement

The studies used for this data analysis were approved by the ethical review committees of the Ghana Health Service, London School of Hygiene and Tropical Medicine, Kintampo Health Research Centre Institutional Ethics Committee, Noguchi Memorial Institute of Medical Research, and The Ghana Food and Drugs Authority. Written informed consent was sought from all mothers whose children participated in the studies.

### 2.2. Site Description

The data analysis was carried out on laboratory data from a study area with a profile presented by Adu-Gyasi et al., 2012 [8]. Briefly, the area is located within the forest-savannah transitional ecological zone in Ghana with perennial malaria transmission [11].* Pf* is the predominating* Plasmodium* species while* P. malariae* and* P. ovale* are in the minority.

### 2.3. Description of Data Sources

The data was obtained from children under 5 years who were recruited into malarial studies carried out by Kintampo Health Research Centre between the periods of October 2008 and March 2011. In the first study, children were enrolled and followed actively every month for two years. The children were referred to the hospital for care whenever they were unwell to determine the incidence of malaria [12]. The second study which was a clinical trial was to determine the effect of providing Micronutrient Powder with or without iron on the incidence of malaria among children under three years living in a high malaria-burden area in Ghana [13].

### 2.4. Blood Sample Collection and Processing

In each of the studies, blood samples were collected by finger-prick into 0.5 mL microtainers containing ethylenediaminetetraacetic acid (K2EDTA-BD, USA) from the participants on their scheduled visit days and on any other day participants visited the health facilities with illness. Samples were collected and transported according to the Standard Operating Procedures (SOPs) established by the Kintampo Health Research Centre. Briefly, participant's finger was rubbed, and the tip was cleaned with disposable alcohol swab and allowed to dry. Safety lancet was used to prick and drops of blood were collected into labelled test-tubes to the required volume. Collected samples were used for full blood count analysis and for making blood smears. Examination of thick and thin smears was carried out as described by Adu-Gyasi et al., 2012 [8].

### 2.5. Estimation of Parasite Density

Parasite densities for all participants were calculated using assumed WBCs count of 5000/*μ*L, 6000/*μ*L, 8000/*μ*L, and 10000/*μ*L of blood. In addition, we used the WBCs reference values established for children aged less than one year (10000/*μ*L of blood) and also for children less than five years (9200/*μ*L) [14].

### 2.6. Data Entry, Cleaning, and Analyses

Data obtained from the database of the malarial studies was checked for completeness and consistency and all queries were resolved. All parasite negative blood slide results were removed before analysis. Analyses were done using STATA (version 12; Stata Corp., TX, USA) and GraphPad PRISM version 5.0 (GraphPad Software, Inc.) particularly for geometric mean and intervals. Geometric means at 95% CI that did not overlap were considered significant.

## 3. Results

Of the 39851 results received over the study period, data of 4944 participant results were consistent and complete for the purpose of our analysis. Of the total, 50.6% (2498/4944) were males and 49.4% (2443/4944) were females.

The mean (±SD) WBCs and GMPD of the 4944 positive samples were 10500/*μ*L (±4.1) and 10644/*μ*L (95% CI 9986/*μ*L to 11347/*μ*L) of blood, respectively. There was no significant difference between the mean (SD) WBCs 10600/*μ*L (4.0) for males and 10500/*μ*L (±4.1) for females (*P* = 0.255). Neither was there a significant difference between the GMPD 11058/*μ*L (95% CI 10114/*μ*L to 12091/*μ*L) of blood for males (Table 1) and 10217/*μ*L (95% CI 9324/*μ*L to 11197/*μ*L) of blood for females (Table 2).

With an assumed WBCs count of 8000/*μ*L, a GMPD of 8679/*μ*L (95% CI, 8140/*μ*L to 9253/*μ*L) of blood was estimated (Table 3).

In using the WBCs reference values established among children in the study area, (10400/*μ*L), a GMPD of 11282/*μ*L (95% CI, 10581/*μ*L to 12029/*μ*L) of blood and a WBCs count of 9200/*μ*L produced a GMPD of 9981/*μ*L (95% CI, 9361/*μ*L to 10641/*μ*L) of blood.

The difference in the GMPD calculated using absolute WBCs compared to densities estimated with assumed WBCs was significantly lower for 8000/*μ*L. However, GMPD for assumed WBCs count of 10.0 × 10^9^/L, WBCs reference values of 10400/*μ*L, and 9200/*μ*L estimated a geometric mean parasite density of 10848/*μ*L (95% CI, 10175/*μ*L to 11567/*μ*L), 11282/*μ*L (95% CI, 10581/*μ*L to 12029/*μ*L), and 9981/*μ*L (95% CI, 9361/*μ*L to 10641/*μ*L) of blood that was not significantly different from estimates obtained with the absolute WBCs (Table 3). The interval of GMPD obtained when an assumed WBCs of 8000/*µ*L, 6000/*µ*L and 5000/*µ*L were used and that from absolute WBCs count did not overlap. However, the intervals of GMPD from absolute WBCs count and that obtained with assumed WBCs counts of 9200/*µ*L, 10000/*µ*L and 10400/*µ*L overlapped showing no significant difference in the GMPD (Table 3).

A correlation analysis of the malaria parasite densities with the WBCs reference value and assumed WBCs compared to the absolute WBCs showed a significant level of correlation (*P* < 0.0001) with all the assumed WBCs count (Table 4).

## 4. Discussion

Malaria parasite density is necessary for patient management. This has become dominated by the convenient but inaccurate assumption of a constant WBCs count of 8000/*μ*L of peripheral blood [3, 15] due to lack of capacity to measure patients absolute WBCs [16].

The mean WBCs count from the participants with* malaria* infection compares with the WBCs reference values, 10400/*μ*L for children less than 1 year and 9200/*μ*L for children up to 5 years [14]. The parasite densities from the WBCs reference values agree with the density obtained from using participant's absolute WBCs.

It is agreed that the best solution in estimating parasite densities would be to use the corresponding absolute WBCs count for each age group [17]. Though there is a significant level of correlation, estimating the parasite density of* Plasmodium *species with the assumed WBCs count of 8000/*μ*L of blood [7], compared to the absolute and reference value WBCs count, would mean underestimating significantly the parasite density of* Plasmodium *species infections for patients in the study area. Therefore, establishing regional based reference WBCs as suggested [17] to estimate parasite densities in malarial infections will be appropriate. This was evident in the fact that the geometric mean parasite density, 11282/*μ*L (95% CI, 10581/*μ*L to 12029/*μ*L) and 9981/*μ*L (95% CI, 9361/*μ*L to 10641/*μ*L) of blood, estimated by using the established WBCs standard values of 10,400/*μ*L and 9200/*μ*L of blood for children less than 1 year and up to 5 years old, respectively, overlaps with the geometric mean parasite density obtained by using the mean absolute WBCs count, 10644/*μ*L (95% CI 9986/*μ*L to 11347/*μ*L) of blood, of the participants. Results obtained when an assumed WBCs count of 10000/*μ*L of blood was used to estimate the parasite density [8] were also consistent with that from the absolute WBCs count of participants. The parasitaemia obtained by the use of the other assumed WBCs count was significantly lower (Table 3) [8].

## 5. Conclusions

Since lack of resources in some settings in Africa makes it difficult to estimate malaria parasite density based on actual WBCs count of patients, where available, the study recommends the use of an established WBCs reference value for a known population and where the reference value could be implied. The reference value established in Kintampo has been predicted as a malaria density estimator when compared with the absolute WBCs of participants. In environments where reference values have not been established, the study affirms the use of an assumed WBCs count of 10000/*μ*L of blood to estimate malaria parasite density as documented [8].

## Figures and Tables

**Table 1:** Comparison of parasite densities using absolute WBCs, assumed WBCs, and WBCs reference value among males of the population.

	Absolute WBCs	WBCs count of 5000/*µ*L	WBCs count of 6000/*µ*L	WBCs count of 8000/*µ*L	WBCs count of 9200/*µ*L	WBCs count of 10000/*µ*L	WBCs count of 10400/*µ*L
Number of observations, *N*	2498	2498	2498	2498	2498	2498	2498
Minimum	10	10	12	16	18	20	21
25% percentile	2040	1050	1260	1680	1932	2100	2184
Median	12497	6798	8158	10877	12509	13597	14141
75% percentile	71645	35750	42900	57200	65780	71500	74360
Maximum	2081035	1705766	2046920	2729226	3138610	3411533	3547994
Mean	68255	35019	42023	56031	64435	70038	72840
Std. deviation	139655	79080	94896	126528	145508	158160	164487
Std. error (mean)	139655	79080	94896	126528	145508	158160	164487
Lower 95% CI of mean	62775	31917	38300	51067	58726	63833	66386
Upper 95% CI of mean	73734	38122	45746	60995	70144	76244	79293
Geometric mean	11058	5596	6715	8953	10296	11191	11639
Lower 95% CI of geo. mean	10114	5116	6139	8186	9414	10232	10642
Upper 95% CI of geo. mean	12091	6120	7344	9792	11261	12240	12730

**Table 2:** Comparison of parasite densities using absolute WBCs, assumed WBCs, and WBCs reference value among females of the population.

	Absolute WBCs	WBCs count of 5000/*µ*L	WBCs count of 6000/*µ*L	WBCs count of 8000/*µ*L	WBCs count of 9200/*µ*L	WBCs count of 10000/*µ*L	WBCs count of 10400/*µ*L
Number of observations, *N*	2443	2443	2443	2443	2443	2443	2443
Minimum	13	10	12	16	18	20	21
25% percentile	1843	957	1149	1531	1761	1914	1991
Median	10325	5375	6450	8600	9890	10750	11180
75% percentile	73851	35866	43039	57385	65993	71731	74600
Maximum	2790960	1993543	2392252	3189669	3668119	3987086	4146569
Mean	70980	38690	46428	61904	71190	77380	80475
Std. deviation	158340	102849	123418	164558	189242	205697	213925
Std. error (mean)	3204	2081	2497	3329	3829	4162	4328
Lower 95% CI of mean	64698	34610	41532	55375	63682	69219	71988
Upper 95% CI of mean	77262	42770	51324	68433	78698	85541	88962
Geometric mean	10217	5243	6292	8389	9647	10486	10906
Lower 95% CI of geo. mean	9324	4783	5739	7653	8800	9566	9948
Upper 95% CI of geo. mean	11197	5748	6897	9196	10576	11495	11955

**Table 3:** Comparison of parasite densities using absolute WBCs, assumed WBCs, and WBCs Reference value.

	Absolute WBCs	WBCs count of 5000/*µ*L	WBCs count of 6000/*µ*L	WBCs count of 8000/*µ*L	WBCs count of 9200/*µ*L	WBCs count of 10000/*µ*L	WBCs count of 10400/*µ*L
Number of observations, *N*	4944	4944	4944	4944	4944	4944	4944
Minimum	10	10	12	16	18	20	21
25% percentile	1947	1013	1215	1620	1863	2025	2106
Median	11316	6081	7298	9730	11190	12163	12649
75% percentile	72777	35780	42936	57248	65835	71560	74423
Maximum	2790960	1993543	2392252	3189669	3668119	3987086	4146569
Mean	69618	36851	44221	58961	67805	73701	76649
Std. deviation	149152	91603	109924	146565	168550	183206	190534
Std. error (mean)	2121	1303	1563	2085	2397	2606	2710
Lower 95% CI of mean	65459	34297	41156	54875	63106	68593	71337
Upper 95% CI of mean	73776	39405	47286	63048	72505	78809	81962
Geometric mean	10644	5424	6509	8679	9981	10848	11282
Lower 95% CI of geo. mean	9986	5088	6105	8140	9361	10175	10581
Upper 95% CI of geo. mean	11347	5783	6940	9253	10641	11567	12029

**Table 4:** Correlation analysis of malaria parasite densities using assumed and reference value WBCs count compared with absolute WBCs.

Variable	Mean	Standard deviation	Minimum	Maximum			
Absolute WBCs	69618	149152	10	2790960			
WBCs of 5000/*µ*L	36851	91603	10	1993543			
WBCs of 6000/*µ*L	44221	109924	12	2392252			
WBCs of 8000/*µ*L	58961	146565	16	3189669			
WBCs of 9200/*µ*L	67805	168550	18	3668119			
WBCs of 10000/*µ*L	73701	183206	20	3987086			
WBCs of 10400/*µ*L	76649	190534	21	4146569			

	Absolute WBCs	WBCs count of 5000/*µ*L	WBCs count of 6000/*µ*L	WBCs count of 8000/*µ*L	WBCs count of 9200/*µ*L	WBCs count of 10000/*µ*L	WBCs count of 10400/*µ*L

Absolute WBCs	1.0000						
WBCs of 5000/*µ*L	0.8825	1.0000					
WBCs of 6000/*µ*L	0.8825	1.0000	1.0000				
WBCs of 8000/*µ*L	0.8825	1.0000	1.0000	1.0000			
WBCs of 9200/*µ*L	0.8825	1.0000	1.0000	1.0000	1.0000		
WBCs of 10000/*µ*L	0.8825	1.0000	1.0000	1.0000	1.0000	1.0000	
WBCs of 10400/*µ*L	0.8825	1.0000	1.0000	1.0000	1.0000	1.0000	1.0000

Significance level of correlation is *P* < 0.0001.

## Limitations of the Study

The ages of all participants were not stratified into groups. This did not make it possible to compare differences at the possible age groups less than 5 years.
